# Cluster randomised trials of prescribing policy: an ethical approach to generating drug safety evidence? A discussion of the ethical application of a new research method

**DOI:** 10.1186/s13063-020-04357-4

**Published:** 2020-06-05

**Authors:** Amy Rogers, Gillian Craig, Angela Flynn, Isla Mackenzie, Thomas MacDonald, Alexander Doney

**Affiliations:** 1 MEMO Research, School of Medicine, University of Dundee, Ninewells Hospital, Dundee, DD1 9SY UK; 2 Health and Clinical Services, School of Medicine, University of Dundee, Ninewells Hospital, Dundee, DD1 9SY UK; 3 Department of Physics, University of Strathclyde, 107 Rottenrow East, Glasgow, G4 0NG UK

**Keywords:** Clinical trials, Cluster randomization, Ethics, Comparative effectiveness

## Abstract

For most chronic medical conditions, multiple medications are available and prescribers often have limited evidence about which therapy is likely to be the most effective and safe for an individual patient. As many patients are exposed every day to medicines that may be less effective than available alternatives, this is of public health importance. Cluster randomised trials of prescribing policy offer an opportunity to rapidly obtain evidence of comparative effectiveness and safety. These trials can pose a low risk to patients and cause minimal disruption to usual care. Despite the potential scientific value of this approach, there remain valid concerns about consent, medication switching and the use of routinely collected data in research. We discuss these concerns with reference to an ongoing pilot study (Evaluating Diuretics in Normal Care (EVIDENCE) - a cluster randomised evaluation of hypertension prescribing policy, ISRCTN 46635087, registered 11 August 2017).

## 1. Background and aims

The World Health Organization (WHO) has highlighted the importance of drug safety research in providing doctors with evidence to inform prescribing decisions [1]. For any medical condition, there may be several licensed treatment options with similar modes of action. Additionally, the evidence used to support licensing applications is usually from randomised controlled trials of limited duration and using non-representative care pathways [2]. External validity is further limited by trial participation being restricted to exclude individuals with comorbidities. Thus, prescribers are faced with a choice between multiple medicines but with limited applicable comparative safety and effectiveness evidence. In the absence of such evidence, prescribing choices are made on the basis of cost, anecdotal experience or non-evidence-based guidelines [3].

Comparative effectiveness research using observational methods can be used to fill these evidence gaps by studying medicines when they are already in use. These studies often include much larger numbers of patients, who may have more complex illness and multimorbidity, for longer follow-up periods, making up for some of the generalisability shortcomings of interventional pre-licensing studies. However, they can be difficult to interpret because of data quality concerns, unmeasured confounders and baseline imbalances of dependent variables, all of which may bias estimates of treatment effect. Observational research is often considered by prescribers to be insufficient to change practice. Newer observational methods – such as propensity score matching, instrumental variables, marginal structural models and sensitivity analyses – aimed at improving causal inference should improve this situation [4–6]. But randomisation, by significantly reducing baseline imbalance, allows researchers to make stronger claims of cause and effect. Post-licensing randomised drug comparisons remain uncommon as there is little incentive for a company to test their product against a competitor’s [7]. Surveillance systems, such as the UK Yellow Card Scheme [8], are designed to detect safety signals post-licensing but rely on individuals recognising events as potential adverse drug effects. Therefore, it is vital for public health that robust research frameworks be developed to monitor, evaluate and compare the safety and effectiveness of medicines after licensing. We describe a cluster randomised study design that could be used within such a framework, when clinically useful evidence from more traditional sources is not available, and consider the ethical implications of this approach.

## 2. Discussion

### 2.1. Cluster randomisation of prescribing policy

Traditional randomised controlled trials, usually considered the gold standard for comparing treatments, are expensive and time-consuming; in 2012 the estimated average cost per patient in a UK trial was £7890 [9]. Using only this type of trial to compare the expanding range of available drugs is not feasible. Cluster randomised trials, where interventions are applied to groups of individuals, are more economical to implement and can be scaled up readily across health services, allowing more efficient and equitable use of finite research resources. A major advantage of cluster randomised trials over individually randomised trials is that the former improve generalisability, as randomisation can include all individuals who would be eligible for an intervention and not just those who choose to participate. Trials using an opt-out recruitment approach, or with a waiver of consent, can study patient populations more representative of people with the condition in question, whereas opt-in only recruitment results in healthier, smaller, and non-representative study populations [10, 11]. These advantages must be balanced against a need for careful statistical planning to take account of intracluster correlation and baseline covariate imbalance [12–14]. A study that produces highly generalisable results may be seen as more directly relevant to practice and more likely to effect change in prescriber behaviour. Cluster randomised trials of prescribing policy are one pragmatic solution to comparing drug treatments that are already licensed and in use within a health-care system but that lack direct comparative effectiveness data. Randomisation is not performed on individuals but on existing groups of individuals, such as hospitals, clinics, or primary care practices. The intervention is prescribing policy applied at the cluster level. This approach to comparative effectiveness evaluation was suggested by Sabin et al. in their discussion of the ethics of cluster randomised trials [15]. The method was tested by McCarren et al. in their report on the feasibility of using cluster randomisation to compare chlorthalidone and hydrochlorthalidone [16], randomly assigning health-care providers to favouring one agent over the other for any new prescriptions of thiazide diuretics. That study was granted a waiver of individual consent to broaden inclusion. The approach was accepted by providers, but over the 9-month study, only 138 patients (at 18 sites) were newly prescribed a thiazide medication. Clearly, applying randomisation to new medication starts only would require a very large number of sites to be randomly assigned to achieve statistical significance for most clinical outcomes. We propose a further development of this method that uses mechanisms for the routine switching of long-term prescribed medications that already exist within the UK National Health Service (NHS).

### 2.2. Prescribing policies and medication switching

Routine changes to prescribing policy are common in the NHS and often occur when there are price differences between similar drugs, supply problems, or newly available evidence. Local, regional or national NHS bodies make decisions on preferred prescribing choices via reimbursement, formularies or guidelines. Individual prescribers in the NHS remain free to prescribe as they see fit, and there are limitations only for very costly or high-risk treatments. Prescribing policy changes can result in changes being made to a patient’s existing routine medications and require no ethical approval or individual patient consent, although a general notification of the change is sometimes provided [17]. These bulk medication changes, referred to as “switches” within the NHS, can be met with suspicion or confusion, and patients sometimes object to changes towards cheaper generic drugs [17]. However, the public is generally accepting of medications being changed to evaluate safety or effectiveness. When asked in a survey how they would react upon receiving a letter informing them of a medication change, 61% of UK respondents said they would be "happy" or they "would not mind" [18]. When the reason for the change was given as “to find out which drug works better”, 67% stated they would be "happy" or they "would not mind" [18].

### 2.3. Example study

We have piloted the methods of a cluster randomised trial of prescribing policy to compare two commonly used diuretics in the management of hypertension, bendroflumethiazide and indapamide. The aim of the pilot was to test the feasibility and acceptability of the intervention and study procedures. Twenty-nine medical practices in Scotland have been randomly assigned to a prescribing policy specifying which study drug should be first-line. Practices agreed to adopt the assigned policy for future prescribing and existing routine prescriptions have been switched accordingly. Doctors remain free to select the most appropriate medication for their patients. All patients eligible for a potential medication switch have been informed by letter of the policy change. The letter explains the reason for any medication changes and directs patients to visit the study website or contact the study team (by telephone or email) to find out more about the study or to opt out of the proposed medication change. De-identified routinely collected prescribing and hospitalisation data will be used to assess post-randomisation prescribing and baseline covariates. In the full study, these data will be used to identify clinically important outcomes, including cardiovascular hospitalisations and mortality.

Cluster trials of medicines are a relatively new concept in health care, and few specific formal ethical and regulatory guidelines are available for researchers. They have attracted criticism over ethical concerns about identification of the participant and the need for individual informed consent [19, 20]. Cluster randomisation of prescribing policy raises additional concerns around medication switching and the use of routinely collected data.

### 2.4. Informed consent

Ethical guidelines such as the Ottawa Statement [21] and those produced by the Council for International Organizations of Medical Sciences (CIOMS) and the WHO have considered cluster trials to be a distinct trial type [22]. One important area of ethical debate is whether individuals affected by interventions applied at the cluster level should be considered participants and whether they should be subject to individual informed consent requirements [23]. In the example study described, it is clear that patients are directly affected by the policy randomisation and that it is feasible for an individual patient to opt out of having their medication changed without affecting the rights of other patients. For this reason, we have considered them to be participants. The primary concern, therefore, is whether formal opt-in individual consent is required in this study design.

Informed consent aims to ensure that an individual is free to choose to participate, has sufficient information about the research, and is capable of providing consent. Individual informed consent is often considered an absolute ethical requirement in clinical research. Indeed, some believe a lack of individual informed consent means that a study cannot ethically proceed [24]. Currently accepted methods of informing patients about research studies are not always effective at achieving these aims [25, 26]. Also, obtaining individual consent can significantly add to the cost and duration of a study [27–30] and may damage the generalisability of findings by restricting recruitment to more motivated and literate participants [31, 32]. The blanket application of ethical guidelines to low-risk situations, such as where licensed medications already in common usage are being compared, has been criticised as hindering valuable research [33–35]. Consequently, there have been discussions about whether there is a need to modify existing consent regulations, such as opt-out consent, for health-care research considered to be of low risk to patients [27, 33]. Another suggestion is a policy of general notification to patients in health-care systems that certain decisions about care may be subject to research [36].

Any requirement to gain individual informed consent in the context of policy randomisation applied at the practice level should be balanced against other ethical concerns. Doctors and patients need evidence to make informed decisions. All health-care decisions require the balancing of potential benefits and risks, but lack of evidence for the relative effectiveness of one drug over another means that choices between treatments cannot be fully evidence-based. Although every prescribing decision made under these conditions carries some risk, health-care providers who engage with cluster randomised prescribing policy studies would be doing so in the best interests of their future patients. Clinicians could even be accused of acting unethically for failing to acknowledge existing uncertainty about the best choice of treatment and not informing a patient of the inherent risk of non-evidence-based treatments [37].

Cluster randomised trials of prescribing policy present an opportunity to carry out societally important research but they must be designed to operate within current ethical and legal frameworks. This is despite the lack of consensus amongst researchers, regulators and ethicists on when individual informed consent to either policy-level interventions or data collection is necessary or essential [20, 38, 39].

While prescribing policies affect groups of patients, we believe that clinicians and their patients should be informed of the reason(s) for policy changes and reserve the right to choose whether to follow a prescribing policy in individual treatment decisions. In this way, the individual autonomy of both prescriber and patient can be preserved. Patients should never be forced into research participation, but, for studies comparing currently accepted standards of care, it may be appropriate that patients simply be informed that research is taking place and be given the right to withdraw from the intervention or from data collection (or both) without affecting their care [27]. The right to withdraw from the intervention in the study method we have described is a scientific trade-off – made to maintain ethical integrity – that will introduce a degree of selection bias.

### 2.5. Routinely collected data

Routine health-care data have great potential for clinical trials with a major advantage to cluster randomised policy evaluation of avoiding disruption to usual care [40]. It has been argued that patients have a moral responsibility to allow access to their data to improve the health-care system for all [35]. However, the use of routinely collected medical data for research does raise additional ethical and regulatory concerns [40]. Potential risks in using routinely collected data include loss of patient confidentiality and loss of patient trust in research [40]. Existing programmes do allow patients some choice about whether their data can be used for reasons not directly related to their own care. For example, the NHS England National Data Opt-out has been exercised by 2.74% of patients [41]. There are currently no mechanisms for patients in the UK to choose which studies may use their data.

## 3. Conclusions

The method we have outlined above could play just one part in a comprehensive medicines safety and effectiveness programme. It is most suitable for comparing long-term outcomes for multiple similar drugs already in widespread use but could be adapted to compare new treatments against existing standards of care (e.g., evaluating a planned introduction of a newly licensed medication). The method would be less suitable for use in health systems where patients and practitioners are not familiar with institutional prescribing policies and routine medication switching. Implementing this methodology on a large scale will require significant efforts to engage prescribers and patients. Each proposed medicine comparison would still need to be considered on a case-by-case basis by an appropriate ethical review body to ensure that the rights of individuals are not unduly limited.

Although this method should improve external validity and does increase the feasibility of large-scale comparative effectiveness research within the NHS, it is not yet known to what extent patients choosing not to accept proposed medication changes might cause problematic selection bias. The results of our pilot study should inform this consideration. This study design is a hybrid between randomised controlled trial and observational methods. Whether this is an acceptable compromise remains a matter for debate.

There is an unmet need for comparative effectiveness research to determine the best medicine for many conditions, and randomised prescribing policy evaluations are an opportunity to efficiently meet this need. This type of trial challenges traditional research ethics and consent standards. While ethical guidelines are important to protect individual patient rights and welfare, facilitating research that could resolve medical uncertainties for society as a whole should be of at least equal concern. From a public health perspective, the current situation, where patients are routinely exposed to medicines that have never been compared directly against each other, is neither clinically nor ethically acceptable. The pilot study we have described above, conducted within the Scottish NHS, proposes one way to address this problem. This study design integrates with local health-care practice with the key difference of policy allocation being actively managed by randomisation and clinical outcomes being formally assessed. Further development of this methodology could allow for comparisons of multiple interventions to take place simultaneously within health-care systems.

## Data Availability

Not applicable.

## Abbreviations

NHS: National Health Service
WHO: World Health Organization
